# “Upcycling” known molecules and targets for drug-resistant TB

**DOI:** 10.3389/fcimb.2022.1029044

**Published:** 2022-10-06

**Authors:** Christine Roubert, Evelyne Fontaine, Anna M Upton

**Affiliations:** Evotec ID (Lyon), Lyon, France

**Keywords:** drug resistance, target, antibiotics, phenotypic screening, golden age of antibiotics

## Abstract

Despite reinvigorated efforts in Tuberculosis (TB) drug discovery over the past 20 years, relatively few new drugs and candidates have emerged with clear utility against drug resistant TB. Over the same period, significant technological advances and learnings around target value have taken place. This has offered opportunities to re-assess the potential for optimization of previously discovered chemical matter against *Mycobacterium tuberculosis* (M.tb) and for reconsideration of clinically validated targets encumbered by drug resistance. A re-assessment of discarded compounds and programs from the “golden age of antibiotics” has yielded new scaffolds and targets against TB and uncovered classes, for example beta-lactams, with previously unappreciated utility for TB. Leveraging validated classes and targets has also met with success: booster technologies and efforts to thwart efflux have improved the potential of ethionamide and spectinomycin classes. Multiple programs to rescue high value targets while avoiding cross-resistance are making progress. These attempts to make the most of known classes, drugs and targets complement efforts to discover new chemical matter against novel targets, enhancing the chances of success of discovering effective novel regimens against drug-resistant TB.

## Introduction

Despite 20 years of new vigor in Tuberculosis (TB) drug discovery, the number of novel compounds that have potential utility against drug resistant TB, and that have successfully advanced to clinical studies, is low. The Target Regimen Profiles for TB, for example those set out by the World Health Organization (WHO, 2016), set a high bar for contributing drugs, aiming for regimens that shorten treatment compared to the standard of care and are improved with respect to route of administration (oral replacing non-oral), tolerability, drug-drug interactions and with a limited need for monitoring. Considering these goals and the desire to de-risk drug candidate properties prior to clinical development, it is understandable that significant attrition is seen across discovery and early development, especially when considering novel chemical series for which precise safety, pharmaceutical and distribution, metabolism and pharmacokinetics (DMPK) profiles are, a priori, unknown. Programs seeking to discover and develop novel treatments suitable for drug-resistant TB face an additional constraint that can limit options when considering novel chemical matter - that is the requirement that the series of interest exhibit very limited or no pre-existing resistance (Miotto et al., 2017; Coll et al., 2018; Walker et al., 2022).

Most TB drug resistance is drug-target related, so significant efforts have been expended towards discovery of modulators of new targets. However, new targets, by definition, are not yet clinically validated. So far, for the small number of development compounds against new targets, the success rate in demonstrating proof of concept efficacy in Early Bactericidal Activity clinical trial trials has been high (Diacon et al., 201). This is likely due to the care that has been taken by teams to evaluate and preclinically-validate new targets, as well as the good overall performance of the tools available to enable prediction of clinical efficacy, including animal models of TB. However, a key desirable property for novel TB treatments is the ability to shorten treatment duration to cure, compared to the standard of care. For this specific property, due to the limited clinical data available, it is more challenging to predict the degree to which a drug against a new target will contribute.

Over recent years, several strategies have emerged, to address the risks inherent in progressing novel chemical matter against novel targets. These involve a second look at what may be already-existing possibilities: to leverage known antibacterial classes for TB (with the advantage of their known safety and DMPK profiles, but lack of pre-existing resistance), and to “rescue” clinically validated TB drug targets (with known clinical efficacy profiles) that have been compromised by resistance. In both cases, this second-life brings together pre-existing opportunities with new technologies and thinking, against drug-resistant TB (Lohrasbi et al., 2018; Bandodkar et al., 2020; Singh and Chibale, 2021).

This perspective summarizes the progress of these approaches to date, together with evident advantages and limitations of these strategies.

## Revisiting known classes and abandoned programs from the golden age of antibiotics

Prior to the renewed efforts of the past 20 years, the last phase of major activity in TB drug discovery was during what is now called “the golden age of antibiotics” (Lewis, 2013). Multiple successful TB drugs emerged from this period, including Streptomycin (1943), para-aminosalicylic acid (1946), isoniazid (INH, 1952), pyrazinamide (PZA, 1952), ethambutol (ETH 1961) and rifampicin (RIF, 1966) (Chakraborty & Rhee, 2015). Many other TB drug research projects began and were then discontinued during this time, often for scientific and/or business reasons. In some cases, based on anti-M.tb activity data available at the time, classes such as beta-lactams were developed for other antibacterial indications but were not progressed for TB. In other cases, compounds were advanced to early clinical studies but were discontinued due to low probability of commercial success judged based on the contemporary landscape and capabilities. A second, new-millennium look at cases of these types has brought new insights to the TB drug target space as well as progression of known classes and molecules re-directed for potential utility against TB.

### Maximizing utility of known classes

Beta-lactams are an exceptionally safe class of antibiotics. They kill bacteria by inhibiting the transpeptidase that catalyzes the final step in cell wall biosynthesis - a source of several clinically validated TB drug targets. However, this class was long considered to be ineffective against M.tb, due to rapid hydrolysis by the M.tb beta lactamase and was never used to treat TB. Therefore, unlike the situation for other cell wall targeting TB drugs, no clinical record of beta-lactam resistance exists. In an example of a successful strategy to look again at a well-known class, evaluated the activity of carbapenems combined with clavulanic acid, a beta-lactamase inhibitor, was evaluated against M.tb, demonstrating potent activity of the meropenem-clavulonate combination (Watt et al., 1992; Huggonet et al., 2009). This opened the door to clinical exploration of this class for TB, with early bactericidal activity of meropenem, administered intravenously combined with amoxicillin–clavulanic acid, demonstrated in 2016 (Diacon et al., 2016). This clinical proof of concept motivated a search for an oral beta-lactam with utility for TB, resulting in the repurposing of the tricyclic beta-lactam Sanfetrinem, cilexetil, the oral prodrug of sanfetrinem, developed by GlaxoSmithKline in the 1990s (Singh et al., 1996; Iavarone et al., 1997). This drug is currently in early development (Phase 2, NCT05388448) following preclinical demonstration of anti-M.tb activity (Ramon-Garcia, 2019). In parallel, in an extraordinary example of the use of pharmaceutical companies’ patrimony in a collaborative approach, an initiative led by the Tuberculosis Drug Accelerator consortium screened about 8900 beta-lactams from GSK, Sanofi, Lilly, and MSD (Gold et al., 2022), looking for safe, *in vivo* active and possibly beta-lactamase inhibitor independent compounds.

In another example of successful revisiting of known classes, spectinamides, semisynthetic analogs of Spectinomycin, which was discovered in 1961 (Mason et al., 1961; Bergy et al., 1961), have demonstrated efficacy against TB in mouse models and MBX-488A has progressed to preclinical development as a potential TB drug. Spectinomycin exhibits poor activity against M.tb (Lee et al., 2014), despite targeting protein synthesis – a validated TB drug target - *via* the 30S subunit of the bacterial ribosome. It is used to treat gonorrheal infections but demonstrates limited tolerability and is dosed with intramuscular injections. The semisynthetic spectinamides, on the other hand, demonstrate improved selectivity as well as more potent anti-M.tb activity by avoiding efflux through the M.tb efflux pump Rv1258c. In a remarkable medicinal chemistry effort, Lee and colleagues optimized the series to avoid the Rv1258c efflux pump resulting in leads that demonstrate significant activity in acute and chronic mouse models of TB and contribute to combinations of TB drugs (Lee et al., 2014; Bruhn et al., 2015; Gonzalez-Juarrero et al., 2021).

Macrolides represent another class of antibacterial protein synthesis inhibitors that were thought to lack potency and utility against TB. This thinking has now been challenged by, for instance, the SEQ-503 macrolide from the Sanofi Natural Product patrimony, discovered in 1962 in Vitry-sur Seine and named after sequana, the seine goddess in the Gallo-Roman religion models (Lair et al., 2015). Optimization of SEQ-503 has given rise to SEQ9 which has a lower MIC (0.6 µM) than previously tested macrolides, for example Clarithromycin (8 µM), and is similarly potent to the more active macrolides reported by Falzari and colleagues (Falzari et al., 2005). Similar to the substituted 11,12 carbazate macrolide reported by Falzari et al, SEQ9 is active in mouse TB models (Lair et al., 2015); another demonstration of a new activity for a revisited class of antibiotics.

### Bringing today’s technologies to yesterday’s discoveries

Beyond rethinking potential utility of known drug classes, like ghosts from the past, compounds discovered in the golden age of antibiotics but not advanced to market can be identified, retrieved and re-addressed with a new view. Such activities have been invigorated in the informatics era by document scanning and digitization of archives, providing the possibility to perform extensive searches of databases to select forgotten compounds for improvement or repurposing. Recent examples include the unexplored cyclohexapeptide natural product Desotamide (Miao et al., 1997), or Wollamides A and B (Khazil et al., 2014), which exhibit antimycobacterial activity including inhibition of intracellular M.tb in murine bone marrow-derived macrophages. These were optimized from 2017 resulting in improved pharmacokinetic properties but no description of vivo efficacy has been reported yet (Asfaw et al., 2017; Asfaw et al., 2018; Khalil et al., 2019) (**Table 1**).

**Table 1:** Compounds described in this review.

Coumpound	Suspected Target	Target Class	Pubmed CID	Reported Minimal Inhibitory Concentration MIC (µM) * author generated data	Efficasacy demonstrated in an animal model results can be limited by adm routes/ poor Pk/animal models	Discovery	TB patients
Isoniazed	InhA	Cell wall	3767	0.3*	Yes	1912	1952
Rifampicin	RNA polymerase	Transcription	135398735	0.09	Yes	1957	1971
Meropenem	L,D-transpeptidase	Cell wall	441130	6.5	Yes	1976	2016
Sanfetrinem	L,D-transpeptidase	Cell wall	71452	5.3	Yes	1976?	2022 Phase 2, NCT05388448
Spectinomycin	30S subunit of the bacterial ribosome	Protein synthesis inhibitor	15541	150	low/no activity	1961	
Spectinamides 1599	30S subunit of the bacterial ribosome	Protein synthesis inhibitor	60173108	3.3	Yes	2014	
Clarithromycin	23S rRNA	Protein synthesis inhibitor	84029	8*	No	1980	
SEQ9	23S rRNA	Protein synthesis inhibitor		0.7	Yes	2015	
Desotamide			181446	NA		1997	
Wollamides B	Unknown		102341742	3		2014	
Viomycin	23S/16S	Translation	135398671	5.8-12		1950	
Capreomycin	23S/16S	Translation	3000502	5.8-12	Yes	1960	1966
Histogranine	Unknown/ATP		16131189	6.9		1993	
Amiclenomycin	Biotin pathway	Metabolism	99594	16		1975	
Pyridomycin	InhA	Cell wall	3037036	0.55-3.0		1953	
CPZEN-45	MraY/murX	Cell wall	674119859	2.3	Yes	2003	Pre-Clinical (Non-GLP)
FNDR 20364	inhibiting ribosome associated Gtpase activity	translation		Unknown	Yes	Unknown	GLP toxicology
Cyclomarin A	ClpC1	proteostasis	10772429			1999	
Pleuromutilin	50S subunit	Translation	9886081	2.2	Yes	1951	
Griselimycin	DnaN	Replication inhibitor	429055	0.09	Yes	1964	1964
Ethionamide	ethA	Cell wall	2761171	4.5*		1956	1965
Pretomanid			456199	0.3*		2000	2019
Kanglemycin A	RNA polymerase	Transcription	6443924	0.36	Yes	2018	
Sorangicin A	RNA polymerase	Transcription	657059	Unknown		1985	
Corallopyronin	RNA polymerase	Transcription	90477824	30		1985	
Fidaxomycin	RNA polymerase	Transcription	10034073	0.24		1987	
PUM	RNA polymerase	Transcription	72792467	inactive		2017	
D-AAP1	RNA polymerase	Transcription		4		2017	
Nargenicin	DnaE1	Cell wall	6326334	12.5		1980	
Compound 22 (Spiropyrimidinetrione)	DNA Gyrase	Replication inhibitor		1.7-5.2	Yes	2015	
VXc-486/SPR720	DNA Gyrase	Replication inhibitor			Yes	2014	Phase 2, NCT04553406
Compound 17 (Thiazolopyridone ureas)	DNA Gyrase	Replication inhibitor		2	Yes	2014	
Gepopidacin (GSK2140944)	DNA Gyrase	Replication inhibitor	25101874	0.38		2015	

CID, pubchem compound identification number; DprE1, decaprenylphosphoryl-β-D-ribose 2’-epimerase (Rv3790); EthA, monooxygenase EthA (Rv3854c); DnaN, DNA polymerase III DnaN (Rv0002); ClpC1, TP-dependent Clp protease ATP-binding subunit ClpC (Rv3596c); InhA, NADH-dependent enoyl-[acyl-carrier-protein] reductase (Rv1484); MmpL3, ransmembrane transporter (Rv0206c); LeuRS, leucyl-tRNA synthetase (Rv0041); DnaE1, DNA polymerase III alpha subunit (Rv1547); DHFR, dihydrofolate reductase DfrA (Rv2763c).

In the meantime, TB drug discovery also benefited from the progresses made in peptide chemistry. After Merrifield’s discovery (Merryfield, 1963) on solid-phase peptide synthesis in 1963 and the introduction of basolabile 9-fluorenylmethyloxycarbonyl (Fmoc) able to protect orthogonal side-chain groups (Carpino and Han, 1970), the automation of the process combined to the use of new types of resins have successfully improved the speed and possibility to explore SAR on natural compounds with total or hemisynthesis (Kimmerlin and Sebach, 2005).

Considering the different mergers of pharmaceutical companies, searching and analyzing the archive (patrimony) and compound libraries of pharma companies, not available to the public, might represent a golden opportunity inherited from the past. This strategy was used by Sanofi with the revival of natural product scaffolds) discovered by Rhône-Poulenc-Rorer in the 1960-1980 era. Griselimycin, discovered in 1964, showed success in treating TB, but with poor ADME properties (Hénazet 1966; Noufflard-Gyu-Noé and Berteaux, 1965). The discovery of its unique mechanism of action (Kling et al., 2015) through DnaN, supported by earlier reports of the effectiveness of Griselimycin against drug-resistant *M. tuberculosis* (Toyohara, 1987) led to a new drug discovery program addressing liabilities of this molecule. Advances in peptide chemistry, as described above, were instrumental in taking the original natural product further to produce the lead hexyl compound.

There are many further examples of TB active compounds uncovered from patent or literature searches that revealed antibiotic activity on M.tb and that have been, or have potential for optimization using modern techniques. These include Viomycin and Capreomycin (Youmans & Youmans, 1951; Bycroft, 1972), Histogranin (Lemaire et al., 1993) Amiclenomycin (Okami et al., 1974; Mann and Ploux, 2006; Dey et al., 2010), Isoxazoline (Tangallapally et al., 2007; Phanumartwiwath et al., 2021), and Pleuromutilin (Kavanagh et al., 1951; Lemieux et al., 2018). In some cases, a close look at such compounds using new technologies has also yielded targets of interest, for example cyclomarin A (Renner et al., 1999; Schmitt et al., 2011; Kiefer et al., 2019), which targets the Clp protease complex and was recently used in a proof of concept for chimeric small-molecule degraders, the bacterial-Proteolysis Targeting Chimeras (BacPROTACs) in M.tb (Morreale et al., 2022).

Many of these golden era natural products suffer from lower oral bioavailability. Newer drug delivery systems and formulation technologies can be brought to bear to improve oral bioavailability or even increase specific targeting, once again harnessing modern technology to make use of known or re-discovered TB actives. New formulation adapted to classical TB drugs in the hope to overcome both toxicity and resistance has been reviewed elsewhere (Singh et al., 2016; Mazlan et al., 2021). Combinations of clinically validated antibiotics, encapsulated in nanoparticles, have been investigated in macrophages and this concept could be opening a new path to combinations of drugs with different PK/PD parameters (Jiang et al., 2022). Furthermore, the recent examples of FNDR 20364 (Working Group for New TB Drugs) or CPZEN-45, a caprazamycin derived compound (Salomon et al., 2013) indicates potential for success using these approaches.

## Approaches to address highly validated drugs and targets compromised by resistance

Besides the possible forgotten classes and missed opportunities from the Golden Age, several antibiotics were indeed developed and successfully used for TB from that time and beyond. Unfortunately, most of these are now compromised by resistance. With proven efficacy, these drugs and their targets are well validated and a variety of approaches have been pursued to “rescue” the targets and the drugs themselves for further use including against resistant TB.

### Rethinking known marketed drug classes for TB

Towards rescuing known TB drugs to which resistance has emerged, a potential “short-cut” or repurposing strategy is to reconsider dosing of efficacious clinical anti-TB drugs, to evaluate whether higher exposures than currently used can safely and effectively treat TB due to infections with Mtb strains resistant to that drug. Indeed, the first line drug RIF could be given at higher doses and although current clinical trials are aiming to shorten treatment of drug sensitive TB, the outcome of a safe higher dose of RIF could also impact MDR TB (RIFASHORT, NCT02581527; HIRIF, NCT01408914). The same thinking has been applied to INH with the INHindsight study, a phase 2A dose-ranging trial of INH for patients with pulmonary MDR-tuberculosis and inhA mutations (Dooley et al., 2020; Wasserman and Furin, 2020). Alternatively, members of the same class as existing TB drugs can be evaluated where added value on moderately resistant clinical strains has been implied. An example is the rifampicin analog, rifabutin, which demonstrates activity on some RIF- resistant clinical strains (Yan et al., 2015; Berrada et al., 2016; Alfarisi et al., 2017).

For drugs to which high level resistance has emerged, such dose optimization is often not an option, and alternative efforts are needed. In a noteworthy example of innovation, utilizing a known marketed drug with a “resistance bypassing” strategy, the potentiation of Ethionamide has been achieved, to overcome its deleterious side effects and resistance. Ethionamide, discovered in 1956, is an intra-bacterial-prodrug that requires bioactivation within M.tb to acquire its antibacterial effect. Screening for “Ethionamide boosters” was conceptualized by the Institut Pasteur of Lille (Willand et al., 2009), and this group and collaborators have conducted fragment-based screening and structure-based optimization efforts (Prevet et al., 2019; Villemagne et al., 2020) towards achieving molecules inactivating repressors of the enzymes responsible for the bioactivation of drugs within M.tb, referred to as Small Molecules Aborting Resistance (SMARt) (Blondiaux et al., 2017) with the subsequent discovery of the phase 1 compound BVL-GSK098 (Working Group on New TB Drugs). Now, this group is actively working on this concept for other intrabacterial-prodrugs with low-level pre-existing resistance that may rise to significant clinical resistance in the future, such as pretonamid (Djaout, 2022).

The discovery of specific pathways and underlying druggable targets involved in M.tb’s adaptation to and the subsequent reduction of efficacy of clinically validated anti-TB drugs might be of great value. Recently, ingenious use of a set of inducible transcription factors strains, the Transcriptional Regulator Induced Phenotype (TRIP), representing most annotated M.tb regulators unraveled new uncharacterized regulons and downstream genes involved in the adaptation to INH (Ma et al., 2021). Understanding these mechanisms may lead to strategies to intervene and reverse such drug tolerance that may be the gateway to drug resistance.

As the non-target mechanisms of TB drug resistance continue to be uncovered, there exists an opportunity to identify and develop modulators of these mechanisms, as potential companion drugs, potentially lending new life to further existing TB drugs.

### How can we rescue compromised validated targets

Another approach to overcoming drug resistance is the identification of novel scaffolds that inhibit the few clinically validated targets of the first and second-line anti-TB drugs, which have already shown efficacy or even treatment-shortening behavior. Indeed, the exploitation of new or modified scaffolds against highly validated targets for which existing TB drugs are compromised by resistance can decrease biological and clinical failure risk associated with pursuing compounds against new targets.

An obvious opportunity towards this aim is classical target-based screenings and these have been conducted against validated TB targets, seeking new chemical matter against targets for which current drugs are compromised by resistance. However, these have not met with much success. Small molecule uptake into and metabolism within Mtb, as well as access to molecular targets in the complex lipid-rich cell wall of mycobacteria, has hindered this effort. Freely accessible algorithms have been developed and can be used to predict mycobacterial cell wall penetration (eg MycPermCheck) based on drug activity (Merget et al., 2013). However, it remains to be seen whether such tools can aid in optimizing whole cell penetration for target-based hits without whole cell activity. Alternative approaches to screening, conducted against validated targets and pathways, but in whole cells, may prove more successful (Abrahams et al., 2012; Bonnett et al., 2016; Naran et al., 2016; Abramovitch, 2018; Evans and Mizrahi, 2018; Johnson et al., 2019; Burke et al., 2020; Smith et al., 2020). However, the following outlines alternative and innovative approaches towards this drug and target “rescue” goal.

Besides target-based screening approaches, repurposing and lead optimization strategies have been applied for molecules inhibiting validated targets in a different way to, and without cross-resistance with, important TB drugs. As an example, treatment shortening behavior has been clinically demonstrated with rifamycins, making them key TB drugs. They target the beta-subunit of the RNA polymerase complex encoded by the rpoB gene and this represents a valuable target. However, it is unclear if RIFs are sterilizing due to their specific binding mode, their physicochemical and excellent pharmacokinetic-pharmacodynamic properties including lesion penetration (Sarathy et al., 2016), or a combination of these.

Efforts have been made to repurpose and optimize RNA polymerase inhibitors discovered to be active in other bacterial species. These have been assessed in M.tb. with moderate success *in vitro* (**Table 1**). Examples are Kanglemycin (Mosaei et al., 2018; Peek et al., 2020; Harbotte et al., 2021) and Sorangicin A (Lilic et al., 2020) as well as compounds binding to different pockets than RIF like Corallopyronin A (Haebich et al., 2009; Boyaci et al., 2019); Fidaxomycin (Kurabachew et al., 2008; Boyaci et al., 2018); PUM (Maffioli et al., 2017) and the small molecule D-AAP1 (Lin et al., 2017). Moreover, much progress has been made in the comprehension of M.tb’s RNAP complex and the design of new biochemical and biophysical assays should soon answer this tricky question (Stefan et al., 2020).

New whole‐cell phenotypic screenings focusing on the global protein synthesis pathway, addressing both RNA polymerase and the ribosome, may be the key of new discoveries in this field (Burke et al., 2020). Indeed, protein synthesis inhibitors have also shown their value as antibiotics (Kavčič et al., 2020) in TB treatment. For instance, oxazolidinones’ contributions to regimen have proven their treatment shortening activity in mouse TB models (Zhao et al., 2014; Xu et al., 2019) as well as in the Nix-TB and ZeNix clinical trial (Conradie et al., 2020; Conradie et al., 2022). Drug discovery efforts to date have mostly focused on finding safer oxazolidinones because there is very little pre-existing resistance for this class.

Inhibition of nucleic acid synthesis by inhibiting M.tb’s type II topoisomerase, responsible for ATP-driven introduction of negative supercoils into DNA, has proven to be a successful strategy for antibacterial drugs. However, high level resistance to fluoroquinolones (FQ), used as second-line TB treatments (moxifloxacin, levofloxacin, and gatifloxacin) is observed throughout targeted bacterial species. Significant efforts, including target-based screens have resulted in new antibacterial drugs that are effective against fluoroquinolone-resistant pathogens. These include Spiropyrimidinetriones which act against type II topoisomerase (Basarab et al., 2022; Govender et al., 2022) as well as compounds inhibiting DNA replication with different modes of action like the aminobenzimidazole, VXc-486, alias SPR720 (Locher et al., 2015); Thiazolopyridone ureas (Kale et al., 2014) and more recently, Gepotidacin analogs, members of the “novel bacterial topoisomerase inhibitors” (NBTIs) (Blanco et al., 2015; Gibson et al., 2018). As deeply reviewed by Reiche et al. (2017), the DNA replication machinery is encoded by essential mycobacterial genes. Thus, other replication inhibitors are being explored including compounds targeting topoisomerase I (Sandhaus et al., 2016; Ekins et al., 2017), DNA polymerase complex (by Nargenicin, targeting DnaE1) (Chengalroyen et al., 2022) or Griselymicin (DnaN) as described above. Whether or not these molecules targeting machinery other than the fluoroquinolone target – i.e. type II topoisomerase – will deliver efficacy similar to that of the fluoroquinolone class – remains to be seen. As discussed in Basarab et al., 2022, fluoroquinolones and other DNA-targeting agents that have a characteristic mode of action, by which they elicit an SOS response, demonstrate enhanced bactericidal killing versus those that do not (and tend towards bacteriostatic behavior). It is to be determined whether specific modes of target engagement and/or specific DMPK properties, can lead to the specific efficacy profile of successful TB drugs such as moxifloxacin.

In addition to these efforts to rescue the targets of RIFs and FQs, multiple programs have sought to identify replacements for INH that inhibit its target. In the end, what may have appeared as the most simple of rescue efforts, was not: INH is a prodrug and most INH-resistant clinical isolates arise from mutations in KatG, an enzyme responsible for INH activation within M.tb. So, compounds that inhibit Enoyl Acyl Carrier Protein Reductase (InhA), an enzyme involved in fatty acid synthesis and mycolic acid biosynthesis, without first requiring KatG activation, should be active against most INH resistant strains. But identifying novel compounds that target InhA – so-called Direct InhA Inhibitors (DIIs)- has faced many hurdles like uncorrelated enzymatic inhibition and Mtb activity, or poor ADMET and PK properties (Rožman et al., 2017) and have not delivered efficacy similar to INH in mice. These DIIs might not recapitulate entirely INH’s mode of action against M.tb. Recent knowledge and inhibitor classes have been extensively reviewed by Prasad et al. (Prasad et al., 2021).

Last but not least, the mysterious antibiotic PZA, a prodrug used as a first line anti-TB drug since the 1980s, will be the most challenging to address, since its mechanism of action is not completely understood. Adding PZA to RIF INH and ETH reduced the treatment time to a TB cure from 9 to 6 months and exploring PZA’s mode of action (Ragunathan et al., 2021) may eventually facilitate efforts to discover new pathways involved in sterilization and treatment shortening.

## Conclusion

With the increasing threat of multidrug-resistant (MDR) and extensively drug-resistant (XDR) TB, multiple innovative efforts have been conducted and are ongoing to identify novel chemical matter against M.tb using a variety of screening approaches that are agnostic to drug target. These aim to discover novel M.tb-active classes and, in addition, have a chance to reveal or pharmacologically validate novel targets (Li et al., 2017; Ballinger et al., 2019; Shetye et al., 2020; Nuermberger et al., 2022) that can be pursued. In addition, recent advances in the understanding of TB target vulnerability and in pharmacological and genetic validation of specific novel targets have highlighted additional targets for further work (Bosch et al., 2021; Koh et al., 2022; Smith et al., 2022). Target-based or pathway-specific screens in whole cell systems show particular promise. Multiple compounds representing the fruits of these approaches have entered the clinical TB pipeline. These are extensively reviewed elsewhere (Dartois and Rubin, 2022; Butler et al., 2022)

In this article, we highlight a different swath of activities in the TB drug discovery field; those that seek to leverage known drugs and drug classes, previously discovered but not optimized leads, or validated drug targets that are compromised by resistance. These activities have met with some success and demonstrate the power of collaborative approaches; of bringing new technologies and innovations to bear on old drugs and compounds, and of careful re-assessment of existing data associated with old drugs and abandoned concepts. It is noteworthy that about a third of the compounds reported to the Stop TB Partnership’s Working Group on New TB Drugs as currently undergoing preclinical or clinical development for TB, have been developed through this type of program.

These approaches may in some cases avoid the considerable costs, time and risks associated with the discovery and clinical development of a totally new chemical entity and/or compounds against a completely novel target. However, potential pitfalls abound – the advantage of known properties and profile of an existing class is balanced by its known liabilities. Compounds from the Golden Age might represent forgotten possibilities but come with the challenges inherent to natural products, including possibly limited optimization opportunities due to the smaller number of skilled natural products chemists involved in TB drug discovery today. Finally, care should be taken in assessing the chance of success when attempting to recapitulate a specific efficacy profile of a known TB drug by seeking a novel modulator of the same target. There is still much to learn regarding the specific mode of target engagement, downstream events and DMPK properties that lead to the precise clinical efficacy profiles observed for TB drugs.

Finally, a new path to bypass resistance might not arise from small drug molecules but from RNA network regulation such as ncRNA modulation (Gerrick et al., 2018) or proteinaceous inhibitors that may disrupt important protein-protein interaction (Sala et al., 2014), or from antimicrobial peptides (Oliveira et al., 2021).

By conducting diverse drug discovery and development efforts that encompass both identification of novel series and targets and the optimization or rescuing of known classes and targets, the field may produce sufficient substrate for novel treatment-shortening regimens that are effective against drug-resistant TB. Directing our energies to all approaches in a balanced, innovative and collaborative manner will undeniably represent new hopes for fighting TB resistance.

## Data availability statement

The datasets presented in this study can be found in online repositories. The names of the repository/repositories and accession number(s) can be found in the article/supplementary material.

## Author contributions

CR and AU have designed and written the article. EF provided scientific advice and knowledge and carefully read and edited of the manuscript. All authors contributed to the article and approved the submitted version.

## Acknowledgments

We acknowledge support from Eric Bacqué for scientific advice and for careful reading of the manuscript.

## Conflict of interest

Authors CR, EF, and AU are employed by Evotec ID Lyon, France (CR and EF) and Evotec, US (AU).

## Publisher’s note

All claims expressed in this article are solely those of the authors and do not necessarily represent those of their affiliated organizations, or those of the publisher, the editors and the reviewers. Any product that may be evaluated in this article, or claim that may be made by its manufacturer, is not guaranteed or endorsed by the publisher.

## Abbreviations

TB, Tuberculosis; WHO, World Health Organization; DMPK, Drug metabolism and pharmacokinetics; M. tb, Mycobacterium tuberculosis; BacPROTACs, Bacterial-Proteolysis Targeting Chimeras; MDR, Multidrug-resistant; XDR, Extensively drug-resistant; InhA, Enoyl Acyl Carrier Protein Reductase; PZA, pyrazinamide; RIF, Rifampicin; INH, Isoniazid; ETH, ethambutol; FQ, fluoroquinolones; CID, Compound identification number.
